# Cadmium Malignantly Transforms Normal Human Breast Epithelial Cells into a Basal-like Phenotype

**DOI:** 10.1289/ehp.0900999

**Published:** 2009-08-13

**Authors:** Lamia Benbrahim-Tallaa, Erik J. Tokar, Bhalchandra A. Diwan, Anna L. Dill, Jean-François Coppin, Michael P. Waalkes

**Affiliations:** 1 Inorganic Carcinogenesis Section, Laboratory of Comparative Carcinogenesis, National Cancer Institute (NCI) at National Institute of Environmental Health Sciences (NIEHS), National Institutes of Health, Department of Health and Human Services, Research Triangle Park, North Carolina, USA; 2 Basic Science Program, SAIC-Frederick, Inc., Frederick, Maryland, USA

**Keywords:** basal-type, breast cancer, cadmium, estrogen receptor, malignant transformation

## Abstract

**Background:**

Breast cancer has recently been linked to cadmium exposure. Although not uniformly supported, it is hypothesized that cadmium acts as a metalloestrogenic carcinogen via the estrogen receptor (ER). Thus, we studied the effects of chronic exposure to cadmium on the normal human breast epithelial cell line MCF-10A, which is ER-negative but can convert to ER-positive during malignant transformation.

**Methods:**

Cells were continuously exposed to low-level cadmium (2.5 μM) and checked *in vitro* and by xenograft study for signs of malignant transformation. Transformant cells were molecularly characterized by protein and transcript analysis of key genes in breast cancer.

**Results:**

Over 40 weeks of cadmium exposure, cells showed increasing secretion of matrix metalloproteinase-9, loss of contact inhibition, increased colony formation, and increasing invasion, all typical for cancer cells. Inoculation of cadmium-treated cells into mice produced invasive, metastatic anaplastic carcinoma with myoepithelial components. These cadmium-transformed breast epithelial (CTBE) cells displayed characteristics of basal-like breast carcinoma, including ER-α negativity and HER2 (human epidermal growth factor receptor 2) negativity, reduced expression of BRCA1 (breast cancer susceptibility gene 1), and increased CK5 (cytokeratin 5) and p63 expression. CK5 and p63, both breast stem cell markers, were prominently overexpressed in CTBE cell mounds, indicative of persistent proliferation. CTBE cells showed global DNA hypomethylation and c*-myc* and k*-ras* overexpression, typical in aggressive breast cancers. CTBE cell xenograft tumors were also ER-α negative.

**Conclusions:**

Cadmium malignantly transforms normal human breast epithelial cells—through a mechanism not requiring ER-α—into a basal-like cancer phenotype. Direct cadmium induction of a malignant phenotype in human breast epithelial cells strongly fortifies a potential role in breast cancer.

Breast cancer is a common disease and a leading cause of cancer deaths in women (Bray et al. 2004; Parkin et al. 2005). However, the etiology of breast cancer remains incompletely defined. Evidence indicates that both endocrine and environmental factors play mechanistic roles in female breast cancer (Bray et al. 2004), and estrogenic hormones are implicated as major determinants of breast cancer risk (Bernstein 2002; Bray et al. 2004). Endogenous estrogens impact normal breast growth and development, increasing proliferation of critical cell populations, whereas exogenous, pharmacologic estrogens and xenoestrogens likely contribute to accumulated breast cancer risk (Bernstein 2002; Bray et al. 2004). However, classical estrogens alone cannot account for all cases of human breast cancer (Coyle 2004).

Cadmium is a toxic metal and common environmental contaminant [International Agency for Research on Cancer (IARC) 1993; Waalkes 2003]. A human lung carcinogen, cadmium has several other target sites in rodents, including tissues considered endocrine sensitive (IARC 1993; Waalkes 2003). Recent data indicate that human cadmium exposure may be associated with female breast cancer (McElroy et al. 2006), although this initial, hypothesis-forming work does not allow for establishment of definitive causality. There are no corollary data showing carcinogenic activity for cadmium in female rodent mammary tissue, but it can cause mammary gland proliferation in mice (Johnson et al. 2003). Additional studies, including *in vitro* cancer model studies, are critical to clarify any role for cadmium in this important and deadly disease.

Cadmium probably acts in all stages of the oncogenic process, and acts through multiple, nonexclusive mechanisms such as oxidative stress, oncogene activation, apoptotic bypass, and altered DNA methylation (Waalkes 2003). Recently, it was proposed that cadmium acts as a metalloestrogen via interactions with estrogen receptor-α (ER-α), stimulating downstream estrogen-related processes (Garcia-Morales et al. 1994; Johnson et al. 2003; Stoica et al. 2000). This has led to fears that cadmium could act as an xenoestrogen in estrogen-related cancers such as breast cancer (Darbre 2006). It is suspected that a critical early event in many breast cancers is constitutive activation of the ER-α (Zhang et al. 2005). Data indicating that human cadmium exposure may be a risk factor in breast cancer (McElroy et al. 2006) support a concern but do not actually address mechanism. Indeed, the theory that cadmium is metalloestrogenic has not been fortified by actual data associating it with acquired malignant phenotype *in vivo*, such as breast tumors in animals, or *in vitro*, such as malignantly transformed breast cells. Other researchers have found that cadmium lacks strong estrogenic activity or actually inhibits ER (Le Guével et al. 2000; Silva et al. 2006). We found little evidence of ER-α activation *in vivo* or *in vitro* by cadmium (Coppin JF, Waalkes MP, unpublished data). It is evident that cadmium can act through various non–estrogen-related mechanisms, and several mechanisms can occur simultaneously. Further, breast cancer is not always a disease that is absolutely estrogen dependent (Coyle 2004).

Given the importance of female breast cancer, the emergence of data indicating that cadmium may be a risk factor and the unresolved proposal that this could occur through a metalloestrogenic mechanism both clearly indicate that additional research is needed, including research using *in vitro* carcinogenesis model systems. Thus, it was our goal to investigate the role of ER in a cell model of cadmium-induced breast cancer. We examined the malignant transformation of human normal breast epithelial MCF-10A cells after chronic, low-level cadmium exposure *in vitro*. MCF-10A cells are frequently used as a normal control in breast cancer studies and are considered negative for ER-α and ER-β, but they can show ER activation with malignant transformation. The capability to directly trigger a malignant phenotype in human breast epithelial cells would significantly fortify emerging data on the human breast as a target of cadmium carcinogenesis.

## Materials and Methods

### Cells and cell culture

MCF-10A cells, derived from normal human breast epithelium and immortalized but nontumorigenic (Soule et al. 1990), were grown in a base medium (MEGM Bullet Kit; Cambrex, East Rutherford, NJ), with all additives supplied in the kit except cholera toxin. Cultures were incubated at 37°C in 5% CO_2_ in a humidified atmosphere and passed weekly. Cells were exposed continuously to 2.5 μM cadmium (CdCl_2_; purity 99%; Sigma, St. Louis, MO) for up to 40 weeks. We used cultures grown in cadmium-free medium as passage-matched controls. Once malignant transformation was established by formation of xenograft tumors, they were designated cadmium-transformed breast epithelial (CTBE) cells.

We used untreated MCF-7 human breast cancer cells as positive controls for ER-α and ER-β protein and SKBR3 human breast cancer cells (Chrestensen et al. 2007) as positive controls for HER2 (human epidermal growth factor receptor 2) protein.

### Matrix metalloproteinase-9

As an indication of malignant phenotype, secreted matrix metalloproteinase-9 (MMP-9) activity was assessed as described (Benbrahim-Tallaa et al. 2005) during cadmium exposure. Activity was measured in conditioned media by zymographic gels, and quantitation was based on control values set at 100%.

### In vitro invasion

We examined the effect of cadmium on *in vitro* invasive ability using a modified Boyden blind-well chamber assay (Tokar and Webber 2005). Data were based on control cells set at 100%.

### Colony formation

We assessed effects of chronic cadmium exposure on cellular ability to form colonies when plated in soft agar as described by Tokar and Webber (2005). Data were normalized to control cells set to 100%.

### Xenograft tumorigenicity

Animal care was provided in accordance with the Guide for the Care and Use of Laboratory Animals (Institute of Laboratory Animal Resources 1996). The animals were treated humanely and with regard for alleviation of suffering. Mice were housed under conditions of controlled temperature, humidity, and light cycle.

For the xenograft study [National Cancer Institute (NCI)-Frederick Animal Facility], 1 × 10^6^ control cells or chronic cadmium-treated (40 weeks) cells were inoculated bilaterally under the renal capsules (50 μL/capsule) of 10 female nude (NCr-*nu*) mice (NCI-Frederick) per cell-treatment group. Mice were palpated twice daily for signs of tumors and killed when tumors developed or at 6 months after inoculation. A complete necropsy was performed, and obvious tumors, both kidneys, and all abnormal tissues were fixed in 10% buffered formalin. Tissues were embedded in paraffin, sectioned, stained with hematoxylin and eosin (H&E), and analyzed by light microscopy.

### Gene expression analysis

We identified gene expression at the protein level by Western blot (Benbrahim-Tallaa et al. 2005). Total protein was isolated and electrophoresed on NuPAGE 4–12% Bis-Tris gel (Invitrogen, Carlsbad, CA) and transferred to nitrocellulose membranes following the manufacturer’s instructions. Immunoblots were performed using antibodies for human k-*ras*, c-*myc*, and ER-β (at 1:1000 dilution; all from Calbiochem, San Diego, CA), CK5 (cytokeratin 5; at 1:1,000 dilution; Abcam, Cambridge, MA), human ER-α (1:100; Calbiochem), HER2 (1:500; Santa Cruz Biotechnology, Santa Cruz, CA), metallothionein (MT1/2; 1:200; Santa Cruz Biotechnology), or ΔNp63 (p63; 1:100; Santa Cruz Biotechnology). We then used horseradish peroxidase–conjugated anti-rabbit secondary antibodies and anti-mouse secondary antibodies (1:5,000; Amersham, Pittsburgh, PA) with the SuperSignal West Pico chemiluminescent substrate (Pierce, Rockford, IL). Signals were visualized by Hyperfilm (Amersham), and densitometric analysis was performed using Quantity One software (Bio-Rad, Hercules, CA). The data were normalized to individual β-actin and adjusted to control as 100%.

We determined gene expression at the transcript level by reverse transcription-polymerase chain reaction (RT-PCR) as described previously (Benbrahim-Talla et al. 2005). The data were normalized to individual β-actin level and adjusted to control as 100%. All primers were synthesized by Invitrogen. The primers were as follows:

β*-actin*

forward: CCCAGATCATGTTTGAGACCT

reverse: GAGTCCATCACGATGCCAGT

*BRCA1* (breast cancer susceptibility gene 1)

forward: GCTCTTCGCGTTGAAGAAGT

reverse: TGTGGAGACAGGTTCCTTGA

c*-myc*

forward: CTCCCTCCACTCGGAAGGA

reverse: CGGTTGTTGCTGATCTGTCTCA

K*-ras*

forward: CCCAGGTTCAAGCGATTCTC

reverse: GAGTGTAGTGCACACGCCTGTAA

CK5

forward: GTAGCAGCTCCAGCGTCAAAT

reverse: TTGGAAGGCAGTGACTTGCA

p63

forward: CCCCAAGCAGTGCCTCTACA

reverse: GGTGAATCGCACAGCATCAA

aromatase (*CYP19A*)

forward: CTGGCCTTTTTCTCTTGGTG

reverse: ATCCCCATCCACAGGAATCT.

### Global DNA methylation

We determined global DNA methylation by the methyl acceptance assay, as described previously (Benbrahim-Tallaa et al. 2005), at 0, 20, and 40 weeks of cadmium exposure.

### Expression of p63 and CK5

Cells were plated on coverglass chamber slides, grown to confluence, and fixed with acetone:methanol (1:1) for 2 min. Cells were then incubated with primary antibodies (1:200 dilution) against ΔNp63 (p63; Santa Cruz Biotechnology) or CK5 (Abcam) for 2 hr, washed with PBS (3 times 15 min), incubated with AlexaFluor 488 and AlexaFluor 569 fluorescent-conjugated secondary antibodies (Molecular Probes; Invitrogen, Carlsbad, CA) for 1 hr, and then washed with PBS (3 times, 15 min). DAPI (4′,6-diamidino-2-phenylindole; 1:1,000) was added for 5–10 min, and cells were rinsed with PBS (3 times, 15 min). Images were immediately taken using a DP72 camera and IX71 microscope (Olympus, Center Valley, PA).

### Tumor ER-α

We used CTBE cell–generated xenograft tumors for immunohistochemical analysis of ER-α protein. For a positive control, we used an ER-α–positive human breast tumor paraffin block (PanTomics, Richmond, CA). We used polyclonal antibody against human ER-α as the primary antibody (at a dilution of 1:1,000) and a streptavidin-conjugated secondary antibody. Antibody binding was visualized with an avid-in-biotin-peroxidase kit (VECTASTAIN Elite ABC Kit; Vector Laboratories, Burlingame, CA) with diaminobenzidine as the chromagen and hematoxylin as a nuclear counterstain. As a control, the primary antibodies were omitted.

### Statistical analysis

All data except tumor incidence are presented as mean ± SE from three or more independent samplings. Significance was determined by Student’s *t*-test, by analysis of variance followed by Dunnett’s multiple comparison test, or by Fisher exact test as appropriate, with *p* ≤ 0.05 considered significant.

## Results

### Cadmium-exposed breast cells acquire a cancer phenotype

**Figure 1:**
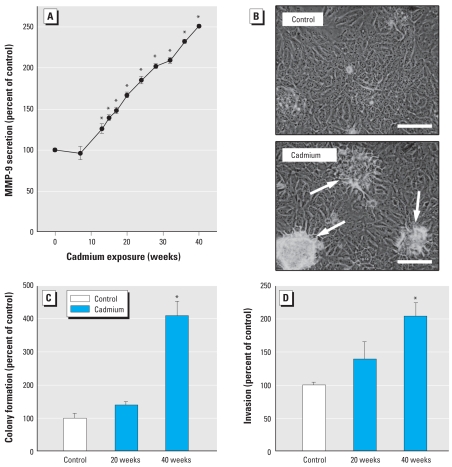
Chronic cadmium exposure induces a cancer phenotype in human breast epithelial cells exposed to 2.5 μM cadmium for up to 40 weeks compared with passage-matched, untreated control cells. (*A*) Active MMP-9 secretion during cadmium exposure. (*B*) Loss of contact inhibition at 40 weeks of cadmium exposure as indicated by formation of foci of cell mounding (arrows; bottom) that were rarely seen in control (top). Bars = 100 μm. (*C*) Increased colony formation in soft agar with chronic cadmium exposure. (*D*) Increased invasive ability with cadmium exposure. Numerical data are expressed as a percentage of control (set to 100%) ± SE. *Significantly different from control.

We assessed the ability of chronic, low-level cadmium to induce transformation in the MCF-10A ER-negative human breast epithelial cell line using various measurements including MMP-9, an enzyme secreted to degrade the extracellular matrix and facilitate tumor cell invasion. A marked, progressive increase in the secretion of active MMP-9 occurred with cadmium exposure (Figure 1A). By 40 weeks of exposure, cadmium-exposed cells also started forming cell mounds when confluent (Figure 1B); this mounding indicates a loss of contact inhibition, which allows cells to continue to divide and form multiple horizontal layers, a common occurrence with cancer cells. Although mounding was common in cadmium-treated cells, it was seldom observed in control cells. Cadmium-treated cells even formed mounds when subconfluent, which we did not observe in controls (data not shown). Cadmium also markedly increased colony formation in soft agar by 40 weeks of exposure (Figure 1C), which is typical of cancer cells and is thought to reflect anchorage-independent growth of tumor-initiating/cancer stem cells (Stingl et al. 2006; Tokar and Webber 2005). Invasive ability was also greatly increased by 40 weeks of cadmium exposure (Figure 1D), a common characteristic of cancer cells.

### Cadmium-exposed breast cells acquire a malignant phenotype

**Figure 2:**
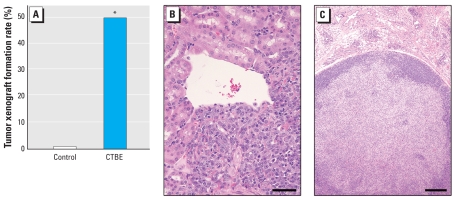
Tumor formation resulting from inoculation of CTBE cells into nude mice. (*A*) Tumor formation rate during 6 months after inoculation of CTBE or control cells under the renal capsules of 10 mice/group. (*B*) Representative section of an anaplastic carcinoma invading the normal kidney, which formed after CTBE inoculation; bar = 50 μm. The tumor is highly aggressive, with areas of epithelial, mesenchymal, and undifferentiated cells. (*C*) A representative metastasis to a peritoneal lymph node of a carcinoma produced by CTBE cell inoculation; bar = 500 μm. *Significantly different from control.

Compelling evidence that cadmium had triggered a malignant phenotype was provided when malignant tumors formed in mice that had been inoculated under the renal capsule with cells chronically exposed to cadmium (40-week exposure) (Figure 2A). The CTBE cells produced highly aggressive carcinoma within as little as 1 month. No tumors arose after inoculation with control cells. CTBE cells produced highly malignant, invasive, anaplastic carcinoma with myoepithelial components containing epithelial, mesenchymal, and undifferentiated cells (Figure 2B). CTBE cell tumors showed metastatic potential, as exemplified by a metastasis to a regional lymph node (Figure 2C). Invasive carcinomas make up approximately 85% of all diagnosed human breast cancers, and regional node metastasis is common with aggressive breast tumors.

### CTBE cells have basal-like malignant breast tumor characteristics

**Figure 3:**
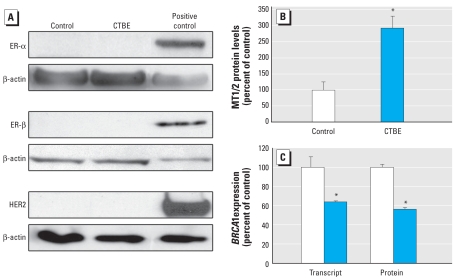
Expression of ER-α, ER-β, HER2, BRCA1, and MT in CTBE cells. (*A*) ER-α, ER-β, and HER2 protein levels showing clear negativity of control MCF-10A and CTBE cells. The positive controls were ER-α- and ER-β-positive MCF-7 cells and HER2-positive SKBR3 cells. Western blots are typical examples of triplicates using β-actin as the loading control and were not quantitated because of the very low protein levels in control and CTBE cells. (*B*) MT protein showing relatively low expression in control but increased expression in CTBE cells. (*C*) BRCA1 protein and transcript in CTBE and control cells. The data for MT and BRCA1 are expressed as a percentage of control (set to 100%; ± SE). *Significantly different from control.

Various breast cancer phenotypes have been defined based on molecular pathology, including the myoepithelial basal-like carcinoma of the breast that is characterized as ER-negative and HER2-negative with increased expression of CK5 and p63 (Fadare and Tavassoli 2007; Yehiely et al. 2006). Indeed, the ER-negative MCF-10A cells remained negative for ER-α and ER-β protein when they became CTBE cells (Figure 3A). ER-α and ER-β proteins were undetectable in CTBE cells compared with a positive control breast cancer cell line (MCF-7). Also, genes downstream of ER-α driven by estrogens were not activated by chronic cadmium in CTBE cells, including pS2 (data not shown). Control and CTBE cells also showed no HER2 protein (Figure 3A) compared with an HER2-positive breast cancer line (SKBR3). In contrast, MT, which is overexpressed in ER-negative breast cancers, was relatively low in control cells but markedly increased in CTBE cells (Figure 3B). Further, MCF-10A cells are considered to have normal BRCA1 function (You et al. 2004), yet BRCA1 expression was suppressed in CTBE cells (Figure 3C).

**Figure 4:**
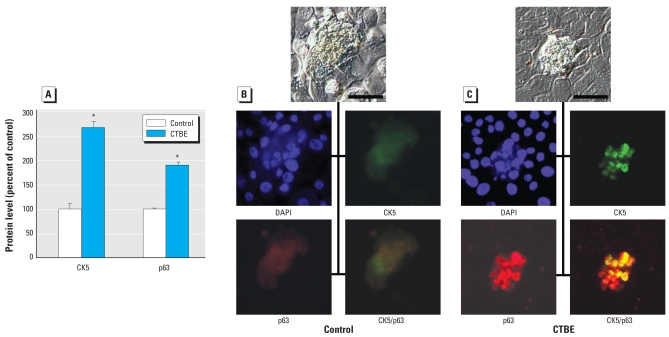
Expression of genes in CTBE cells typical for basal-like human breast cancer phenotype and/or breast stem cells. (*A*) Protein expression for CK5 and p63 expressed as a percentage of control. Fluorescent microscopy was used to determine localization of stem cell marker protein expression in (*B*) control cells and (*C*) CTBE cells. Expression of CK5 (green) and p63 (red) were clearly co-localized to foci of cell mounding (yellow) in CTBE cells and, in comparison, barely detectable in similar structures from control cells. DAPI was used as a nuclear stain to show similar number of viable cells. Top images (gray) are relief contrast. Bars = 25 μm. *Significantly different from control.

Both CK5 and p63 were overexpressed in CTBE cells (Figure 4A). CK5 and p63 are considered stem cell markers in breast tissue, and p63 may act as an oncogene. Foci of cell mounding, common in CTBE cells but rare in controls, indicate cells with loss of contact inhibition that maintain active proliferation. When we assessed foci for p63 and CK5, we found little or no expression in an uncommon foci from control cells (Figure 4B), but the much more commonly occurring CTBE cell mound foci showed intense expression for both p63 and CK5 protein in association with the mound (Figure 4C).

### CTBE cells acquire characteristics of aggressive malignant breast cancer cells

**Figure 5:**
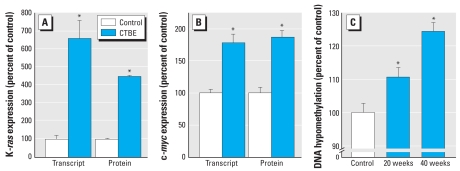
Oncogene activation and global DNA hypomethylation during acquired malignant phenotype in CTBE cells. (*A*) K*-ras* expression. (*B*) c*-myc* expression. (*C*) DNA methylation. Protein or transcript data are expressed as percentage of control (set to 100% ± SE). Note broken scale in (*C*). *Significantly different from control.

Compared with control cells, CTBE cells showed marked increases of both K*-ras* (Figure 5A) and c*-myc* (Figure 5B), oncogenes that are commonly overexpressed in aggressive breast cancers (Eckert et al. 2004; Jamerson et al. 2004). In breast cancers, DNA hypomethylation decreases progressively as tumor grade worsens (Agrawal et al. 2007), and CTBE cells showed a significant and progressive increase in global DNA hypomethylation with cadmium exposure (Figure 5C).

### Xenograft tumors remain ER-α negative

**Figure 6:**
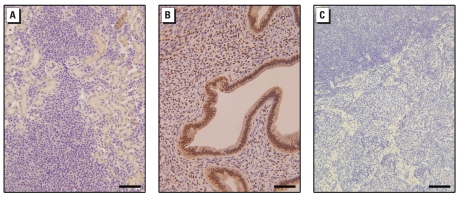
Immunohistochemical analysis for ER-α in xenograft tumors produced by CTBE cell inoculation (*A, C*) and a positive control (*B*). (*A*) Example of a tumor formed by inoculation of CTBE injection, showing minimal ER-α protein in the cells of the tumor. (*B*) A commercially available, known ER-α-positive human breast carcinoma showing strong, nuclear staining (brown). (*C*) A lymph node metastasis from a CTBE-formed tumor showing minimal ER-α protein. Bars = 100 μm.

The remarkable cellular expansion in going from the tissue culture environment to a xenograft tumor could provide a stimulus for acquired expression of genes not seen *in vitro*, such as ER-α. However, analysis of CTBE-induced xenograft tumors showed minimal ER-α protein in the tumor cells (Figure 6A) compared with strong nuclear staining in a human breast carcinoma known to be ER-α positive (Figure 6B). A lymph node metastasis from a CTBE-formed tumor also showed minimal ER-α protein (Figure 6C).

### Aromatase in CTBE cells

Cadmium may have indirectly provided MCF-10A cells with estrogen via increased aromatase activity. However, transcript analysis indicated that CTBE cells showed no higher levels (145 ± 34% of control; *n* = 3) than passage-matched control cells (100 ± 21%).

## Discussion

Environmental factors may account for a large portion of human breast cancers, perhaps approaching 60% (Coyle 2004). Established risk factors such as exogenous estrogens account for a significant portion of this risk (Bernstein 2002; Bray et al. 2004) but do not explain the remainder (Coyle 2004). The increasing incidence and geographic variation of human breast cancer has begun to focus attention on the etiologic potential of other environmental factors (Coyle 2004). Cadmium, a common environmental pollutant, may be such a factor (McElroy et al. 2006) and is noteworthy as a biologically persistent and cumulative metal (IARC 1993; Waalkes 2003). Unusually high levels of cadmium are found in human breast tissue, perhaps indicating specific binding (Antilia et al. 1996), although interindividual levels vary widely. Cadmium was linked to human breast cancer in a recent population-based, case–control study (McElroy et al. 2006). Based on urinary cadmium, both breast cancer risk and tumor aggressiveness increased with increasing exposure (McElroy et al. 2006). This is consistent with our data, where cadmium *in vitro* both induced malignant transformation and produced highly aggressive cells, as the molecular phenotype of the CTBE cells equates to a cancer of poor prognosis (Fadare and Tavassoli 2007; Yehiely et al. 2006). The direct triggering of an acquired malignant phenotype by cadmium in human breast epithelial cells strongly supports the emerging epidemiologic data indicating a role for cadmium in human breast cancer (McElroy et al. 2006).

This present study demonstrated that ER-negative human breast epithelial cells undergo transformation with chronic cadmium exposure. However, CTBE cells remain ER-negative after acquisition of malignant phenotype *in vitro* and even after production of xenograft tumors *in vivo*. Indeed, cadmium produced an apparent basal-like breast cancer phenotype, including ER negativity, HER2 negativity, reduced BRCA1 expression, and increased expression of p63 and CK5, all noteworthy characteristics of basal-like human breast cancer phenotype (Fadare and Tavassoli 2007; Liu et al. 2008; Ribeiro-Silva et al. 2005; Yehiely et al. 2006). Basal-like breast cancers clinically show both poor relapse-free and poor survival rates (Fadare and Tavassoli 2007; Yehiely et al. 2006), and the anaplastic xenograft tumors formed with CTBE cells are consistent with an aggressive tumor with poor prognosis. One mechanism proposed for cadmium is that it acts through actions at ER-α that mimic estrogens, thereby chronically activating pathways that predispose to estrogen-related cancer (Garcia-Morales et al. 1994; Johnson et al. 2003; Stoica et al. 2000). The MCF-10A cells used in this study can be treated in various ways to undergo transformation with the emergence of stimulated ER-α expression as the probable basis for the malignant conversion (Shekhar et al. 1998; Zhang et al. 2005). MCF-10A cells can show activation of genes not seen in basal-like breast cancer phenotypes, such as HER2, with acquired malignant potential (Li et al. 2004). MCF-10A cells are fully capable of reversing their ER negativity during acquired malignant phenotype (Shekhar et al. 1998; Zhang et al. 2005). A key early event in estrogen-dependent breast cancers is activation of ER-α, which can occur with MCF-10A cell transformation (Zhang et al. 2005). Yet, in our model, MCF-10A cells were ER-negative at the onset, remained so *in vitro* after cadmium-induced malignant transformation, and continued to be ER-negative after forming xenograft carcinomas. Cadmium has a variety of possible carcinogenic mechanisms, but from this work it appears unlikely that metalloestrogenic actions through ER were a major factor. Nonetheless, it is possible that cadmium may have metalloestrogenic effects in some instances, and a recent epidemiologic study associated dietary cadmium with endometrial cancer, another site considered estrogen-related (Akesson et al. 2008). However, assumption of mechanism without clear and compelling data may be unwarranted with carcinogens like cadmium, which clearly has multiple possible mechanisms (IARC 1993; Waalkes 2003).

Several studies have shown that human breast tissue concentrates cadmium, and this is exaggerated in cancerous tissue (Antila et al. 1996; Ionescu et al. 2006; Rydzewska et al. 2004; Strumylaite et al. 2008). The metal-binding protein MT avidly binds cadmium and likely accounts for its long tissue-residence time (Cherian et al. 2003). In humans, breast tumor MT overexpression is associated with a poorer prognosis (Jin et al. 2004). A remarkably clear correlation exists in breast tumors between MT overexpression and poor ER expression (El Sharkawy and Farrag 2008), indicating that increased MT may be another basal-like phenotype marker. Tissues often accumulate cadmium associated with MT (Cherian et al. 2003). Thus, whatever mechanisms may operate in the breast, this MT overproduction would toxicokinetically favor cadmium-induced tumor formation by enhancing the metals accumulation.

Both p63 and CK5 expression were markedly increased in CTBE cells. CK5 and p63 are both considered basal-like breast carcinoma markers (Fadare and Tavassoli 2007; Yehiely et al. 2006) and markers for breast stem cells (Boecker and Buerger 2003; Ribeiro-Silva et al. 2005). It appears that p63 functions to preserve adult breast stem cells, facilitating replication and regeneration, possibly by restricting proliferation from an undifferentiated state (Ribeiro-Silva et al. 2005). Similarly, CK5-positive cells likely represent undifferentiated adult stem cells with potential to differentiate into glandular or myoepithelial cells (Boecker and Buerger 2003). CTBE cells show increased expression of both p63 and CK5, particularly in areas of cell mounding (active proliferation), indicating the overproduction of stemlike cells that have lost appropriate differentiation capacity during malignant transformation. An emerging hypothesis is that breast stem cells are critical targets of carcinogens and that blocked differentiation is likely a major pathway to cancer (Dontu et al. 2005). The fact that CTBE cells overexpress stem cell markers and produce a poorly differentiated, aggressive anaplastic xenograft carcinoma is consistent with this hypothesis. Reduced expression of BRCA1 also strongly correlates with overexpression of both CK5 and p63 (Ribeiro-Silva et al. 2005). *BRCA1* is considered to be a breast tumor suppressor gene, and reduced expression or loss of function is associated with ER-negative basal-type breast cancers (Liu et al. 2008; Ribeiro-Silva et al. 2005). Accumulating data indicates that BRCA1 regulates stem/progenitor cell fate in the breast (Liu et al. 2008; Ribeiro-Silva et al. 2005), and loss of function or suppressed *BRCA1* expression may lead to dysregulated stem cell self-renewal or differentiation leading to basal-type breast carcinomas (Liu et al. 2008). Loss of *BRCA1* function can cause the accumulation of genetically unstable breast stem cells, providing critical targets for further carcinogenic events (Liu et al. 2008). Thus, CTBE cells showed both p63 and CK5 over-expression together with a significant loss of BRCA1 expression and ER negativity, all consistent basal breast cancer phenotype (Liu et al. 2008; Ribeiro-Silva et al. 2005), which may indicate a loss of differentiation capacity during the acquisition of basal malignant phenotype.

Further studies are needed to elucidate the mechanisms by which cadmium may cause breast cancer. However, in the present study, cadmium malignantly transformed a breast epithelial cell, producing various molecular hallmarks of a basal-like breast cancer, including ER negativity. Thus, actions for cadmium as a metalloestrogen in this case are unlikely. It is possible that cadmium acted by producing altered DNA methylation status, thereby altering expression of key genes, including oncogenes, as seen in prior work with other cell transformation systems (Qu et al. 2005; Takiguchi et al. 2003). It also appears that cadmium transformation distorted stem cell population dynamics, a common occurrence in oncogenesis (Wicha et al. 2006). Defining the exact mechanism of action for cadmium in the present case will require additional work. Regardless of the precise mechanism, the direct triggering of malignant phenotype by cadmium in human breast epithelial cells unambiguously supports a role for the metal in human breast cancer.
